# Mastectomy Alone or with Immediate Breast Reconstruction: Trend, Precipitating Factors, Patients Reported Outcome, and Oncologic Safety Analysis with and without Propensity Score Matching from 3759 Mastectomy Patients

**DOI:** 10.1007/s00266-025-04762-7

**Published:** 2025-03-06

**Authors:** Yi-Yuan Lee, Hung-Wen Lai, Antonio Muñoz Guevara, Jorge Torres Maldonado, Hui-Yu Lin, Chin-Jung Feng, Bing-Fang Hwang, Shih-Lung Lin, Hsin-I Huang, Fong-Cing Siao, Shou-Tung Chen, Dar-Ren Chen, Chiu-Ying Chen

**Affiliations:** 1 Department of Public Health, China Medical University, No. 100, Section 1, Jingmao Road, Beitun District, Taichung, 406040 Taiwan, ROC; 2 Minimally Invasive Surgery Research Center, Changhua Christian Hospital, Changhua, Taiwan, ROC; 3 Endoscopic and Oncoplastic Breast Surgery Center, Changhua Christian Hospital, Changhua, Taiwan, ROC; 4 Division of General Surgery, Changhua Christian Hospital, Changhua, Taiwan, ROC; 5 Comprehensive Breast Cancer Center, Changhua Christian Hospital, 135 Nanxiao Street, Changhua, 500 Taiwan, ROC; 6 Kaohsiung Medical University, Kaohsiung, Taiwan, ROC; 7 Division of Breast Surgery, Yuanlin Christian Hospital, Yuanlin, Taiwan, ROC; 8 School of Medicine, Chung Shan Medical University, Taichung, Taiwan, ROC; 9 School of Medicine, National Yang Ming Chiao Tung University, Taipei, Taiwan, ROC; 10 Instituto Nacional de Enfermedades Neoplásicas, Faculty of Human Medicine - Cayetano Heredia University, Lima, Peru; 11 Division of General Surgery, Department of Surgery, Breast Center, Cardinal Tien Hospital, Xindien District, New Taipei, Taiwan, ROC; 12 College of Medicine, School of Medicine, Fu Jen Catholic University, New Taipei, Taiwan, ROC; 13 Division of Plastic and Reconstructive Surgery, Department of Surgery, Taipei Veterans General Hospital, Taipei, Taiwan, ROC; 14 Comprehensive Breast Health Center and Division of General Surgery, Department of Surgery, Taipei Veterans General Hospital, Taipei, Taiwan, ROC; 15 Department of Occupational Safety and Health, College of Public Health, China Medical University, Taichung, Taiwan, ROC; 16 Division of Plastic and Reconstructive Surgery, Department of Surgery, Changhua Christian Hospital, Changhua, Taiwan, ROC; 17 Department of Information Management, National Sun Yat-sen University, Kaohsiung, Taiwan, ROC

**Keywords:** Immediate breast reconstruction (IBR), Implant, Oncoplastic reconstructive breast surgeon, Magnetic resonance imaging (MRI), Mastectomy, Propensity score matching (PSM)

## Abstract

**Background:**

In the current study, we surveyed the trend of breast cancer operations in the past two decades and compared mastectomy alone or with immediate breast reconstruction (IBR) with the measurement of the outcome reported by patients and oncologic safety evaluation.

**Methods:**

A retrospective study surveyed the trends in breast cancer surgery methods at a single institution between January 2000 and December 2021. Clinical manifestations, outcomes, patient-reported outcome measures, and oncologic safety evaluations between mastectomy alone or with IBR were analyzed, with and without propensity score matching (PSM).

**Results:**

The trend of breast cancer operations showed that breast-conserving surgery (BCS) and mastectomy with IBR were increasing while mastectomy alone was decreasing. Among the 3759 patients who underwent mastectomies, 1091 (29%) patients had mastectomy with IBR while 2668 (71%) received mastectomy alone. In multivariate analysis, age less than 45 years, breast magnetic resonance imaging before surgery, luminal A subtype, nipple-sparing mastectomy, oncoplastic reconstructive breast surgeon, and high-volume surgeon were important independent factors for mastectomy with IBR. Mastectomy with IBR was associated with better patient-reported cosmetic results than mastectomy alone and comparable to BCS. After PSM and a median follow-up of 106.1 months, there was no difference in Kaplan-Meier survival curve analysis between patients who underwent mastectomy alone or mastectomy with IBR in terms of locoregional recurrence, distant metastasis or overall survival.

**Conclusions:**

Mastectomy with IBR demonstrated better reported cosmetic outcomes and comparable oncologic safety compared to mastectomy alone. Independent factors promoting IBR were identified, which could help increase the breast reconstruction rate.

**Level of Evidence III:**

This journal requires that authors assign a level of evidence to each article. For a full description of these Evidence-Based Medicine ratings, please refer to the Table of Contents or the online Instructions to Authors www.springer.com/00266

## Introduction

Mastectomy, which results in devastating cosmetic outcomes, has long been the primary surgical therapy for breast cancer. Breast-conserving surgery (BCS) has become increasingly common in surgical treatment for single small early breast cancer due to its favorable cosmetic results and acceptable oncologic safety [1–4]. Breast reconstruction with an autologous flap or prosthesis could restore body imaging in breast cancer patients, improve quality of life (QoL), and be an alternative to mastectomy alone when performed immediately [5–8].

Breast screening programs, neoadjuvant chemotherapy, and advancements in surgical techniques, such as oncoplastic breast surgery or conservative mastectomies, have changed the surgical treatment of breast cancer [2, 4]. Advanced skin-sparing mastectomy (SSM) and nipple-sparing mastectomy (NSM) have dramatically increased the incidence of immediate one-stage breast reconstruction, especially implant-based breast reconstruction [9–13].

However, criticism and misunderstanding regarding the oncologic safety of immediate breast reconstruction (IBR) remain one of the main limitations of its indication in women who underwent mastectomy or refused to receive IBR [14, 15]. Identifying variables that facilitate IBR during mastectomy and recognizing factors that place these patients in willingness will help surgeons provide patients with a safe and appropriate reconstruction method after mastectomy [16–20].

In the present study, our objective was to evaluate the change in surgical methods for patients after mastectomy alone, BCS, or with IBR [21–23] during the last 20 years in our institution. Furthermore, the differences in perioperative parameters between patients who underwent mastectomy alone and patients who underwent mastectomy with IBR and factors related to breast reconstruction were evaluated [6, 11, 22, 23]. Finally, we would analyze the oncologic safety of mastectomy combined with IBR compared to mastectomy alone.

## Materials and Methods

### Patients

From January 2000 to December 2021, patients who received breast cancer operations at Changhua Christian Hospital (CCH), a tertiary medical center in central Taiwan, were retrospectively recovered from a prospectively collected breast cancer surgery database. The data collection included clinicopathologic characteristics of patients, types of mastectomies, methods of breast reconstructions, operative time, blood loss, length of hospital stay, and significant complications. All data was collected by specially trained nurses through chart review and subsequently confirmed by the principal investigator (HWL).

**Fig. 1:**
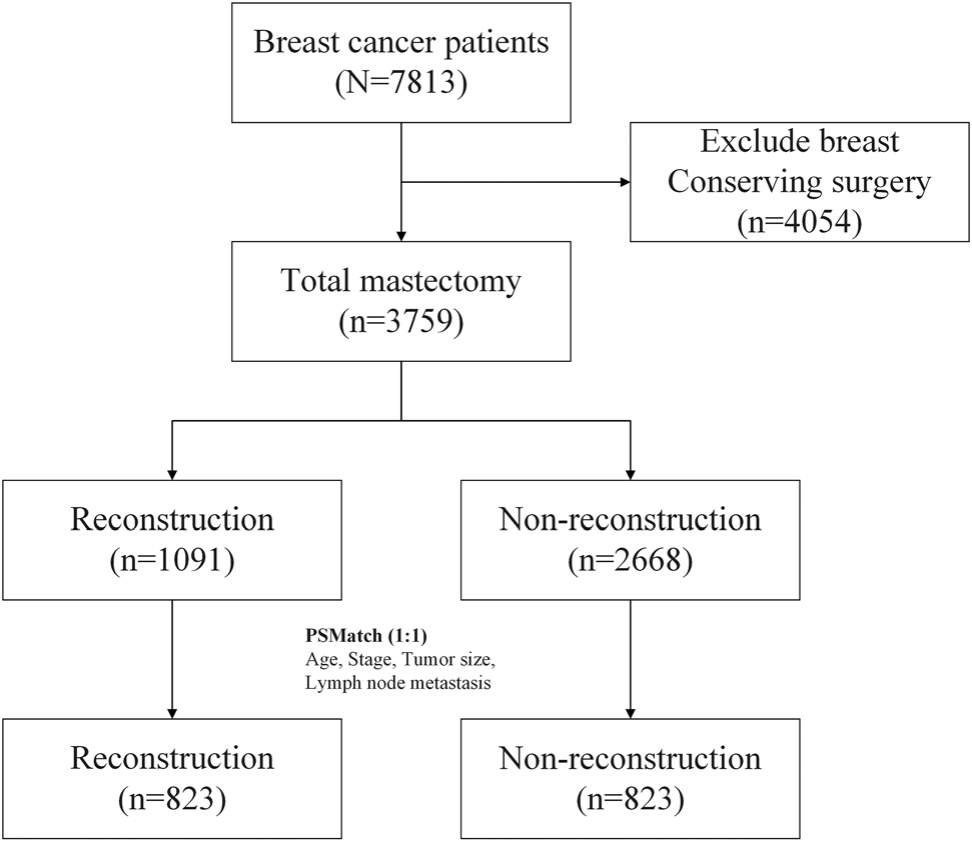
Flow chart of study design. During the study period, a total of 7813 breast cancer patients received surgical treatment at our institution, and 4054 patients received breast conserving surgery (BCS) while 3759 received mastectomies. Among these received mastectomies, 1091 patients underwent mastectomy with immediate breast reconstruction (IBR) and 2668 received mastectomy alone. A case-control comparative study was also conducted using the propensity score matching (PSM) method to decrease bias due to patients’ selection.

During the study period, a total of 7813 breast cancer patients received surgical treatment at our institution. Of these, 4054 patients underwent breast-conserving surgery (BCS), while 3759 received mastectomies. Among those who received mastectomies, 1091 patients underwent mastectomy with IBR, and 2668 received mastectomy alone. The patient selection and enrollment process are shown in Fig. 1.

### Comparison of Mastectomy Alone or with IBR

The perioperative parameters (operation time, blood loss, and hospital stay) and oncologic safety (margin-involved rate, locoregional recurrence, distant metastases, and overall survival) of mastectomy with IBR and mastectomy alone were analyzed and compared. A case-control comparative study was also conducted using the propensity score matching method (PSM) [24–27] to decrease bias due to patient selection.

The differences between patients who received mastectomy with breast reconstruction and those who underwent mastectomy alone were compared to identify factors affecting breast reconstruction. An oncoplastic reconstructive breast surgeon (ORBS) was defined as a breast surgeon who both performed breast cancer surgery and carried out breast reconstruction. A high-volume surgeon was defined as a surgeon who performs breast cancer surgeries on more than 100 patients per year.

For the oncological safety evaluation, we analyzed the rate of positive surgical margin involvement, locoregional recurrence, distant metastasis, disease-free survival, and overall survival. Surgical margin involvement was defined as a tumor on ink [28–30]. Locoregional recurrence was defined as the reappearance of cancer at the operative site. Distant metastasis was defined as any recurrence in distant organs. Overall survival was defined as the time from diagnosis to death from any cause. Adjuvant chemotherapy and radiotherapy were given to patients based on recommendations of current breast cancer guidelines. The incidence of recurrence or death from breast cancer was ascertained at the most recent follow-up, which ended in April 2022.

This study was approved by the Institutional Review Board of CCH (CCH IRB No.: 220217). Written informed consent pertaining to the use of clinical records was obtained from each participant. All patients provided written informed consent.

### Reconstruction Method

Breast reconstruction can be performed using prosthetic methods (with a saline implant, cohesive gel implant, or tissue expander). Autologous breast reconstruction includes the use of transverse rectus abdominis myocutaneous (TRAM) flaps, latissimus dorsi (LD) flaps, or composite flaps (which combine autologous tissue and implants). The type of breast reconstruction is determined through a shared decision-making process between the patient and the treating physicians [31, 32].

### Patient-Reported Outcome Measurements of Aesthetic Results

Patient-reported outcome measures (PROMs) of aesthetic outcomes and quality of life (QoL) were compared between patients who underwent mastectomy alone and those who received IBR. The Breast Q, a validated questionnaire, was used in this study to assess the following domains: satisfaction with breasts, psychosocial well-being, sexual well-being, and physical well-being [33–38].

The wound-related questionnaire is specific to minimal access breast surgery (MABS). A 10-question questionnaire is commonly used to assess PROM in MABS studies [11, 23]. The present study used the same questionnaire to evaluate patient satisfaction with mastectomy and IBR procedures.

### Quality of Life (QoL) Evaluation

QoL was evaluated by recording postoperative pain and resumption of daily activities, with the range of motion, utility, and function of the upper extremity as surrogate indicators. Pain in the upper extremities and breast/chest region was evaluated with a visual analogue scale ranging from 0 (minimum) to 10 (maximum). The range of motion and the utility and function of the upper extremity of the diseased side were also evaluated using self-report questionnaires ranging from 0 (minimal utility) to 10 (maximum range of motion).

### Statistical Analysis

Baseline data were analyzed using chi-square tests (categorical variables) or Student’s t-tests (continuous variables). Associations between mastectomy without reconstruction and mastectomy with reconstruction patients were analyzed using the chi-square test and reported as numbers and proportions. Data are expressed as the mean ± standard deviation (SD) and were compared using the two-sample t-test. Univariate and multivariate logistic regression was performed to assess clinical and pathological factors predisposing patients to breast reconstruction. The matching of the propensity score was used to decrease baseline bias. The propensity score was estimated using the logistic regression model.

All statistical tests were two-tailed, with a significance level (α) set at 0.05. A *p*-value less than 0.05 was considered to indicate statistical significance. The statistical analyses were conducted using SAS version 9.4 (SAS Inc., Cary, NC, USA).

## Results

From January 2000 to December 2021, 7813 breast cancer patients underwent surgery at CCH, 4054 (51.9%) underwent BCS, and 3759 (41.8%) underwent mastectomy. Among the 3759 patients who underwent a mastectomy, the average age was 53.7 ± 11.9 years. The mean tumor size was 3 cm, and 14.3% of the patients had multifocal/multicentric lesions. Lymph node metastasis was found in 41.5% (1470/3759) of the patients. Mastectomies with IBR were performed in 1091 (29%) patients, while 2668 (71%) received mastectomy alone. NSM was performed in 1014 (24.9%) patients. The clinicopathological manifestations of 3759 mastectomies patients were summarized in Table 1.

**Table 1:** Patients demographic of mastectomy alone or with immediate breast reconstruction (IBR) before and after propensity score matching (PSM)

	All	Before PSM			After PSM		
		IBR			IBR		
		Yes (n = 1091)	No (n = 2668)	*P*-value	Yes (n = 823)	No (n =823)	*P*-value
Age^*^, y	53.7 ± 11.9	46.7 ± 9.1	56.5 ± 11.7	< 0.01	47.1 ± 8.9	48.2 ± 8.4	0.01
Age < 45	913 (24.3)	480 (44.0)	433 (16.2)	< 0.01	336 (40.8)	294 (35.7)	0.10
45 ≦ Age < 60	1773 (47.2)	520 (47.7)	1253 (47.0)		421 (51.2)	456 (55.4)	
60 ≦ Age	1073 (28.5)	91 (8.3)	982 (36.8)		66 (8.0)	73 (8.9)	
BMI^*^	24 ± 4	23.1 ± 3.5	24.4 ± 4.1	< 0.01	23.3 ± 3.5	23.8 ± 4.2	< 0.01
Location				< 0.01			< 0.01
Right	1810 (48.2)	578 (53.0)	1232 (46.2)		447 (54.3)	387 (47.0)	
Left	1949 (51.8)	513 (47.0)	1436 (53.8)		376 (45.7)	436 (53.0)	
Pathology tumor size^*^, cm	3.0 ± 2.5	3.0 ± 2.7	3.0 ± 2.4	0.98	2.8 ± 2.4	2.9 ± 2.4	0.79
MRI (NA = 41)				< 0.01			< 0.01
Yes	1965 (52.9)	829 (76.5)	1136 (43.1)		661 (80.6)	381 (46.7)	
No	1753 (47.1)	255 (23.5)	1498 (56.9)		159 (19.4)	435 (53.3)	
Lymph node operations				< 0.01			< 0.01
ALND	914 (28.1)	161 (16.8)	753 (32.8)		127 (16.1)	221 (28.7)	
SLNB	1783 (54.8)	610(63.5)	1173 (51.1)		501 (63.5)	417 (54.2)	
SLNB+ALND	559 (17.2)	189(19.7)	370 (16.1)		161 (20.4)	131 (17.0)	
Lymph node stage				< 0.01			0.58
N0	1993 (60.9)	651 (65.7)	1342 (58.9)		517 (62.8)	518 (62.9)	
N1	771 (23.6)	235 (23.7)	536 (23.5)		211 (25.6)	194 (23.6)	
N2	315 (9.6)	66 (6.7)	249 (10.9)		60 (7.3)	70 (8.5)	
N3	192 (5.9)	39 (3.9)	153 (6.7)		35 (4.3)	41 (5.0)	
Grade				< 0.01			< 0.01
I	438 (13.0)	184 (19.4)	254 (10.5)		157 (19.5)	107 (13.4)	
II	1942 (57.6)	516 (54.5)	1426 (58.8)		440 (54.7)	458 (57.4)	
III	993 (29.4)	247 (26.1)	746 (30.8)		208 (25.8)	233 (29.2)	
Stage^*^				< 0.01			0.96
0	485 (15.2)	193 (20.0)	292 (13.1)		138 (16.8)	132 (16.0)	
I	752 (23.6)	249 (25.7)	503 (22.6)		226 (27.5)	230 (27.9)	
II	1287 (40.3)	374 (38.7)	913 (41.1)		333 (40.5)	327 (39.7)	
III	587 (18.4)	134 (13.9)	453 (20.4)		119 (14.5)	128 (15.6)	
IV	79 (2.5)	17 (1.8)	62 (2.8)		7 (0.9)	6 (0.7)	
Subtype				< 0.01			< 0.01
HER2	416 (14.7)	93 (10.7)	323 (16.5)		73 (10.0)	116 (17.1)	
LuminalA	1114 (39.4)	389 (44.8)	725 (37.0)		332 (45.4)	253 (37.4)	
LuminalB1	544 (19.3)	187 (21.5)	357 (18.2)		164 (22.4)	126 (18.6)	
LuminalB2	439 (15.5)	121 (13.9)	318 (16.2)		99 (13.5)	118 (17.4)	
TNBC	312 (11.0)	78 (9.0)	234 (12.0)		64 (8.7)	64 (9.5)	
Nipple sparing mastectomy				< 0.01			< 0.01
Yes	1014 (29.4)	715 (66.7)	299 (12.6)		541 (66.8)	127 (16.8)	
No	2439 (70.6)	357 (33.3)	2082 (87.4)		269 (33.2)	628 (83.2)	
ER				< 0.01			< 0.01
Positive	2467 (71.1)	763 (77.9)	1704 (68.5)		636 (78.6)	554 (69.3)	
Negative	1002 (28.9)	217 (22.1)	785 (31.5)		173 (21.4)	246 (30.8)	
PR				< 0.01			0.06
Positive	2278 (65.8)	688 (70.6)	1590 (63.9)		574 (71.1)	535 (66.8)	
Negative	1185 (34.2)	287 (29.4)	898 (36.1)		233 (28.9)	266 (33.2)	
HER-2				< 0.01			< 0.01
Positive	980 (31.4)	225 (26.3)	755 (33.3)		180 (24.7)	250 (34.5)	
Negative	2144 (68.6)	630 (73.7)	1514 (66.7)		548 (75.3)	475 (65.5)	
Ki-67				0.02			0.06
≦ 20	988 (55.4)	383 (58.9)	605 (53.4)		345 (61.0)	190 (54.8)	
> 20	795 (44.6)	267 (41.1)	528 (46.6)		221 (39.0)	157 (45.2)	
Lymph node metastasis^*^				0.01			1.00
Yes	1470 (41.5)	356 (35.9)	1114 (43.7)		307 (37.3)	307 (37.3)	
No	2073 (58.5)	637 (64.1)	1436 (56.3)		516 (62.7)	516 (62.7)	
Nipple invasion				<0.01			<0.01
Yes	697 (19.6)	138 (13.8)	559 (21.8)		112 (14.1)	168 (21.1)	
No	2863 (80.4)	861 (86.2)	2002 (78.2)		683 (85.9)	628 (78.9)	
Multifoci/multicentric				0.96			0.90
Yes	430 (14.3)	134 (14.4)	296 (14.3)		119 (15.5)	102 (15.3)	
No	2575 (85.7)	799 (85.6)	1776 (85.7)		647 (84.5)	565 (84.7)	
Radiotherapy				0.66			0.79
Yes	679 (31.8)	145 (31.0)	534 (32.1)		110 (31.3)	147 (30.4)	
No	1455 (68.2)	323 (69.0)	1132(67.9)		242 (68.8)	337 (69.6)	
Surgeon				<0.01			<0.01
ORBS	518 (13.8)	299 (27.5)	219 (8.2)		217 (26.4)	56 (6.8)	
Other surgeons	3233 (86.2)	789 (72.5)	2444 (91.8)		605 (73.6)	763 (93.2)	
Surgeon volume				<0.01			<0.01
High volume	3190 (85.0)	1020 (93.8)	2170 (81.5)		767 (93.3)	678 (82.8)	
Low volume	561 (15.0)	68 (6.3)	493 (18.5)		55 (6.7)	141 (17.2)	

BMI, body mass index; SLNB, sentinel lymph node biopsy; ALND, axillary lymph node dissection; MRI, magnetic resonance imaging; ORBS, oncoplastic reconstructive breast surgeon; ER, estrogen receptor; PR, progesterone receptor; HER-2, human epidermal receptor type 2; TNBC, triple negative breast cancer

### Peri-operative Difference Between Mastectomy Alone or with IBR

Compared with mastectomy alone, patients who underwent mastectomy with IBR tended to have a longer operation time (267 ± 146 min vs. 140 ± 52 min, *P* < 0.01), and more blood loss (77 ± 87 ml vs. 26 ± 5 ml, *P *< 0.01), longer hospitalization (5.9 ± 2.2 days vs. 3.6 ± 1.5 days, *P *< 0.01), a higher risk of delayed wound healing (5.5% vs. 1.4%, *P *< 0.01), and a lower risk of infection (1.6% vs. 4.4%, *P *< 0.01). The risk of seroma or hematoma formation was not significantly different between two groups (Table 2).

**Table 2:** Peri-operative parameters for patients received mastectomy alone or with immediate breast reconstruction (IBR) with and without propensity score matching (PSM)

	Before PSM			After PSM		
	IBR			IBR		
	Yes (n = 1091)	No (n = 2668)	*P*-value	Yes (n = 823)	No (n = 823)	*P*-value
*Peri-operative parameters*						
Total operation time (mins)	267.0 ± 145.7	139.6 ± 52.2	< 0.01	268.4 ± 146.1	144.0 ± 59.4	< 0.01
Total blood loss (ml)	76.5 ± 86.9	26.2 ± 50.9	< 0.01	74.7 ± 84.7	22.5 ± 28.6	< 0.01
Hospital stays (day)	5.9 ± 2.2	3.6 ± 1.5	< 0.01	5.8 ± 2.1	3.6 ± 1.2	< 0.01
Delayed wound healing			< 0.01			< 0.01
Yes	28 (5.5)	22 (1.4)		20 (5.3)	4 (0.9)	
No	479 (94.5)	1570 (98.6)		354 (94.7)	462 (99.1)	
Seroma formation needing aspiration			0.17			0.64
Yes	23 (4.5)	98 (6.1)		22 (5.9)	24 (5.1)	
No	486 (95.5)	1496 (93.9)		352 (94.1)	443 (94.9)	
Blister formation small region						-
Yes	0 (0.0)	0 (0.0)	–			
No	509 (100.0)	1593 (100.0)		374 (100.0)	467 (100.0)	
Hematoma formation			0.70			0.84
Yes	5 (1.0)	19 (1.2)		5 (1.3)	7 (1.5)	
No	504 (99.0)	1575(98.8)		369 (98.7)	460 (98.5)	
Infection			< 0.01			< 0.01
Yes	8 (1.6)	69 (4.4)		6 (1.6)	15 (3.2)	
No	498 (98.4)	1513 (95.6)		365 (98.4)	449 (96.8)	
*Oncologic safety evaluation*						
Margin involved			0.02			0.69
Yes	51 (5.1)	84 (3.4)		38 (4.7)	33 (4.2)	
No	954 (94.9)	2367 (96.6)		778 (95.3)	745 (95.8)	
Recurrence			0.03			0.06
Yes	119 (11.8)	236 (9.4)		90 (11)	68 (8.3)	
No	889 (88.2)	2286 (90.6)		726 (89)	752 (91.7)	
Metastasis			< 0.01			0.71
Yes	132 (13.1)	486 (19.3)		103 (12.6)	108 (13.2)	
No	874 (86.9)	2028 (80.7)		714 (87.4)	713 (86.8)	
Survival			< 0.01			0.08
Yes	1019 (93.4)	2231 (83.6)		769 (93.4)	750 (91.1)	
No	72 (6.6)	437 (16.4)		54 (6.6)	73 (8.9)	

### Types of Breast Reconstructions Performed

**Fig. 2:**
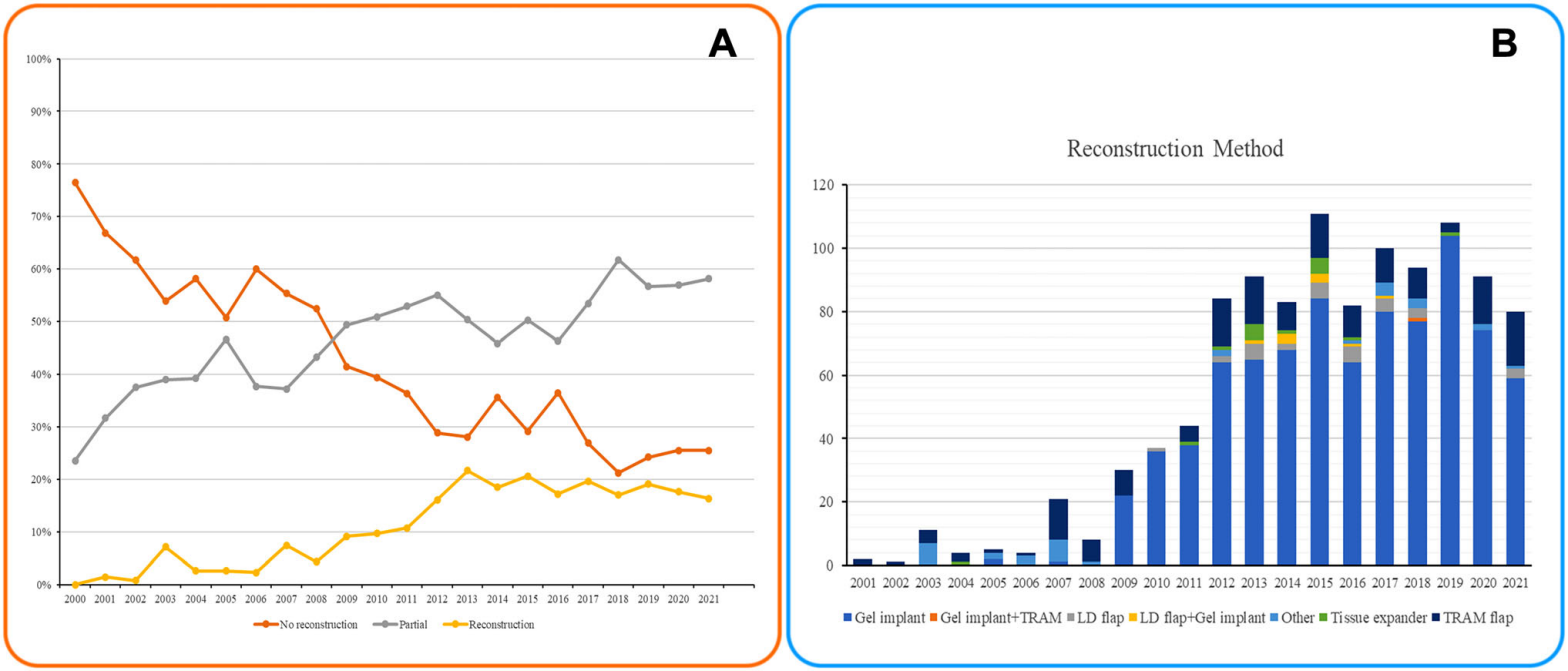
**A** Compares the proportion of patients who receive breast conserving surgery (BCS) and mastectomy with immediate breast reconstruction (IBR) and mastectomy alone. There has been an increase in BCS, a decrease in mastectomy alone, and a gradual increase in mastectomy with IBR. **B** The reconstruction method, by Gel implant, transverse myocutaenous abdominal (TRAM) flap, latissimus dorsi (LD) flap, tissue expander for two stage reconstruction surgery, LD flap with gel implant and Gel implant with TRAM. The trend of the reconstruction method varied from year to year, from 2000 to 2021

During the 20-year study period, the proportion of patients who underwent BCS or mastectomy with IBR increased while the proportion of patients who underwent mastectomy alone decreased gradually (Fig. 2A). Most (77%) of the patients received direct gel implant reconstructions, followed by TRAM flap 15%, LD flap 3%, and with the others 5%. The trend of the reconstruction method varied from year to year. From 2003 to 2007, the TRAM flap was the most widely used method for breast reconstruction. After 2009, gel implant breast reconstruction was the most common reconstruction method (Fig. 2B).

### Factors Related to Breast Reconstruction

Compared to patients who received mastectomy alone, patients who underwent mastectomy and IBR tended to be younger (46.7 ± 9.1 vs. 56.5 ± 11.7, *P *< 0.01), lower body mass index (23.1 ± 3.5 vs. 24.4 ± 4.1, *P *< 0.01), right sided (53% vs. 46%, *P *< 0.01), received pre-operative magnetic resonance imaging (MRI) (76.5% vs. 43.1%, *P *< 0.01), node negative breast cancer (65.7% vs. 58.9%, *P *< 0.01), less axillary lymph node dissection (16.8% vs. 32.8%, *P *< 0.01), higher grade I (19.4% vs. 10.5%, *P *< 0.01), more early stage (0+1) breast cancer (45.7% vs. 35.7%, *P *< 0.01), higher luminal A subtype (44.8% vs. 37%, *P *< 0.01), higher NSM ratio (66.7% vs. 12.6%, *P *< 0.01, Table 1).

In multivariate analysis for IBR mastectomies, age less than 45 years, preoperative breast MRI performed (odds ratio, OR = 3), intrinsic luminal A subtype (OR = 0.8), NSM (OR = 8), ORBS (OR = 1.5), and high-volume surgeon (OR = 2.5) were found to be significantly independent factors related to breast reconstructions (Table 3).

**Table 3:** Univariate and multivariate analyses of clinical and pathologic factors related to mastectomy with immediate breast reconstructions (IBR)

N=3759 (IBR: Yes = 1091, No = 2668)	Univariate analysis			Multivariate analysis		
	OR	95% CI	*P*-value	OR	95% CI	*P*-value
Age, y						
45 ≦ Age < 60 versus Age < 45	0.4	0.32-0.44	< 0.01	0.3	0.25–0.42	< 0.01
60 ≦ Age versus Age < 45	0.1	0.07-0.11	< 0.01	0.1	0.05–0.10	< 0.01
BMI	0.9	0.90-0.94	< 0.01			
Pathology tumor size, cm	1.0	0.97-1.03	0.98			
MRI (Yes versus No)	4.3	3.65-5.03	< 0.01	3.0	2.34–3.95	< 0.01
Grade (II, III versus I)	0.5	0.39-0.60	< 0.01			
Stage (III, IV versus 0, I, II)	0.6	0.50-0.75	< 0.01			
Subtype (other versus Luminal A)	0.7	0.62-0.85	< 0.01	0.8	0.62–0.99	0.04
Lymph node metastasis (Yes versus No)	0.7	0.62-0.84	< 0.01			
Nipple sparing mastectomy (Yes versus No)	14.0	11.70-16.62	< 0.01	8.0	6.28–10.22	< 0.01
Nipple invasion (Yes versus No)	0.6	0.47-0.70	< 0.01			
Radiotherapy (Yes versus No)	1.0	0.76-1.19	0.66			
ORBS versus other surgeons	4.2	3.49-5.12	< 0.01	1.5	1.11–2.12	< 0.01
High volume surgeon versus low volume surgeon	3.4	2.62-4.44	< 0.01	2.5	1.66–3.63	< 0.01

BMI, body mass index; MRI, magnetic resonance imaging; ORBS, oncoplastic reconstructive surgeon; OR, odds ratio; CI, confidence interval

### Patient Satisfaction with the Breast Q Score

According to the analysis of patient satisfaction with the Breast Q score, patients who received mastectomy alone had lower scores than patients with mastectomy and reconstruction (Table 4). Patients who underwent mastectomy and reconstruction had a higher patient satisfaction score than mastectomy alone, and had satisfaction score comparable to those who underwent BCS. Patients who underwent implant-based breast reconstruction had similar satisfaction as did those who underwent autologous breast reconstruction but had significantly shorter wound lengths (6.5 ± 3.2 vs. 18.7 ± 10.2, *P *< 0.01, Table 5). These results were reflected in the increasing number of implant-based breast reconstructions compared to autologous breast reconstructions in the past decade (Fig. 2B).

**Table 4:** Patients reported outcome measurements between mastectomy alone or with immediate breast reconstruction and breast conserving surgery

	BCS (n = 280)	Total Mastectomy		*P*-value
		IBR		
		Yes (n = 323)	No (n = 150)	
Pre-operative breast satisfaction score	2.8 ± 0.8	2.8 ± 0.8	2.6 ± 0.8	< 0.01
Modified Breast Q used at CCH	22.2 ± 5.4	22.2 ± 6.0	13.1 ± 4.1	< 0.01
Satisfaction with Breast (Breast Q)	57.6 ± 12.7	58.5 ± 14.2	50.5 ± 11.8	< 0.01
Social well-being (Breast Q)	65.5 ± 17.4	65.0 ± 17.5	53.9 ± 13.8	< 0.01
Sexual well-being (Breast Q)	47.8 ± 20.3	48.8 ± 18.7	32.8 ± 20.4	< 0.01
Physical well-being (Breast Q)	31.8 ± 17.5	33.4 ± 17.5	30.3 ± 20.5	0.21
If you could choose again, would you choose the same operation again				
Yes	230 (82.1)	244 (75.8)	116 (77.3)	0.30
No	11 (3.9)	20 (6.2)	11 (7.3)	
Unsure	39 (13.9)	58 (18.0)	23 (15.3)	
Wound length	5.9 ± 5.5	7.7 ± 5.8	9.4 ± 5.6	< 0.01
Upper arm pain index	1.1 ± 1.9	1.4 ± 2.2	1.4 ± 2.1	0.21
Chest and breast pain index	1.4 ± 1.9	1.5 ± 2.0	1.3 ± 2.0	0.63
Shoulder range of motion	8.1 ± 2.9	7.2 ± 3.2	7.5 ± 3.1	< 0.01
Upper arm and limb usage in daily life	8.6 ± 2.4	7.8 ± 2.9	7.9 ± 2.8	< 0.01

	BCS (n = 280)	Total Mastectomy +Reconstruction (n = 323)	*P*-value
Pre-operative breast satisfaction score	2.8 ± 0.8	2.8 ± 0.8	0.15
Modified Breast Q used at CCH	22.2 ± 5.4	22.2 ± 6.0	0.98
Satisfaction with Breast (Breast Q)	57.6 ± 12.7	58.5 ± 14.2	0.42
Social well-being (Breast Q)	65.5 ± 17.4	65.0 ± 17.5	0.74
Sexual well-being (Breast Q)	47.8 ± 20.3	48.8 ± 18.7	0.51
Physical well-being (Breast Q)	31.8 ± 17.5	33.4 ± 17.5	0.27
If you could choose again, would you choose the same operation again			
Yes	230 (82.1)	244 (75.8)	0.15
No	11 (3.9)	20 (6.2)	
Unsure	39 (13.9)	58 (18.0)	
Wound length	5.9 ± 5.5	7.7 ± 5.8	< 0.01
Upper arm pain index	1.1 ± 1.9	1.4 ± 2.2	0.09
Chest and breast pain index	1.4 ± 1.9	1.5 ± 2.0	0.56
Shoulder range of motion	8.1 ± 2.9	7.2 ± 3.2	< 0.01
Upper arm and limb usage in daily life	8.6 ± 2.4	7.8 ± 2.9	< 0.01

IBR, immediate breast reconstruction; BCS, breast conserving surgery; CCH, Changhua Christian Hospital

**Table 5:** Patients reported outcome measurements between in mastectomy patients with different reconstruction methods

	Total (n = 323)	Reconstruction Methods		*P*-value
		Flap (n = 33)	Implant (n = 290)	
Pre-operative breast satisfaction score	2.8 ± 0.8	2.8 ± 0.8	2.7 ± 0.8	0.46
Modified Breast Q used at CCH	22.2 ± 6.0	21.5 ± 5.9	22.2 ± 6.0	0.51
Satisfaction with Breast (Breast Q)	58.5 ± 14.2	59.8 ± 4.2	58.3 ± 14.2	0.57
Social well-being (Breast Q)	65.0 ± 17.5	66.2 ± 17.0	64.9 ± 17.6	0.69
Sexual well-being (Breast Q)	48.8 ± 18.7	52.5 ± 18.3	48.4 ± 18.7	0.23
Physical well-being (Breast Q)	33.4 ± 17.5	35.5 ± 20.6	33.2 ± 17.1	0.46
If you could choose again, would you choose the same operation again				0.72
Yes	244 (75.8)	23 (71.9)	221 (76.2)	
No	20 (6.2)	3 (9.4)	17 (5.9)	
Unsure	58 (18.0)	6 (18.8)	52 (17.9)	
Wound length	7.7 ± 5.8	18.7 ± 10.2	6.5 ± 3.2	< 0.01
Upper arm pain index	1.4 ± 2.2	1.3 ± 2.0	1.4 ± 2.2	0.79
Chest and breast pain index	1.5 ± 2.0	1.2 ± 1.6	1.5 ± 2.1	0.42
Shoulder range of motion	7.2 ± 3.2	7.5 ± 3.1	7.2 ± 3.3	0.59
Upper arm and limb usage in daily life	7.8 ± 2.9	7.9 ± 2.7	7.7 ± 3.0	0.74

	Nipple-sparing		*p*-value	Non-nipple-sparing		*P*-value
	Flap (n = 13)	Implant (n = 251)		Flap (n = 20)	Implant (n = 39)	
Pre-operative breast satisfaction score	2.7 ± 0.8	2.7 ± 0.8	0.90	3.0 ± 0.8	2.9 ± 0.7	0.71
Modified Breast Q used at CCH	23.4 ± 6.2	22.9 ± 5.8	0.76	20.3 ± 5.6	18.2 ± 5.5	0.17
Satisfaction with Breast (Breast Q)	58.9 ± 14.2	58.7 ± 14.8	0.96	60.4 ± 14.5	55.9 ± 9.7	0.22
Social well-being (Breast Q)	67.8 ± 13.5	65.3 ± 17.1	0.61	65.2 ± 19.2	62.4 ± 20.4	0.62
Sexual well-being (Breast Q)	55.8 ± 18.6	48.2 ± 18.6	0.15	50.4 ± 18.3	49.9 ± 19.5	0.93
Physical well-being (Breast Q)	39.1 ± 19.7	32.8 ± 16.8	0.20	33.3 ± 21.3	35.4 ± 19.0	0.70
If you could choose again, would you choose the same operation again			1.00			0.95
Yes	10 (83.3)	196 (78.1)		13 (65.0)	25 (64.1)	
No	0 (0.0)	10 (4.0)		3 (15.0)	7 (17.9)	
Unsure	2 (16.7)	45 (17.9)		4 (20.0)	7 (17.9)	
Wound length	10.3 ± 4.4	6.1 ± 3.0	< 0.01	24.1 ± 9.2	8.7 ± 3.5	< 0.01
Upper arm pain index	1.2 ± 1.9	1.4 ± 2.3	0.71	1.4 ± 2.2	1.5 ± 2.1	0.81
Chest and breast pain index	1.2 ± 2.0	1.5 ± 2.1	0.55	1.3 ± 1.4	1.5 ± 2.0	0.57
Shoulder range of motion	7.6 ± 3.0	7.2 ± 3.3	0.68	7.5 ± 3.2	6.9 ± 3.4	0.59
Upper arm and limb usage in daily life	8.2 ± 2.4	7.7 ± 3.0	0.55	7.7 ± 2.9	7.9 ± 2.6	0.76

CCH, Changhua Christian Hospital

### Oncologic Safety Evaluation

Before PSM, the margin involvement rate was 5.1% for mastectomy with IBR versus 3.4% for mastectomy alone (*P* = 0.02); after PSM, the margin involvement rate in the mastectomy with IBR group was 4.7% versus 4.2% for mastectomy alone group (*P* = 0.69). During a mean of 112.1 ± 60.2 months of follow-up, the local recurrence rate was 11.8% in patients who underwent IBR mastectomy versus 9.4% in patients who received mastectomy alone before PSM (*P* = 0.03, Table 2). However, after PSM, there was no difference in the local recurrence rate (11% vs. 8.3%, *P* = 0.06). Before PSM, patients with mastectomy and IBR had a lower rate of distant metastasis (13.1% vs. 19.3%, *P* <0.01), and after PSM, there was no difference (12.6% vs. 13.2%, *P* = 0.071) in terms of distant metastasis. Before PSM, the overall survival rate (93.4% vs. 83.6%, *P *< 0.01) favored patients with mastectomy and IBR, but after PSM, there was no overall survival difference between these two groups (93.4% vs. 91.1%, *P* = 0.08; Table 2).

**Fig. 3:**
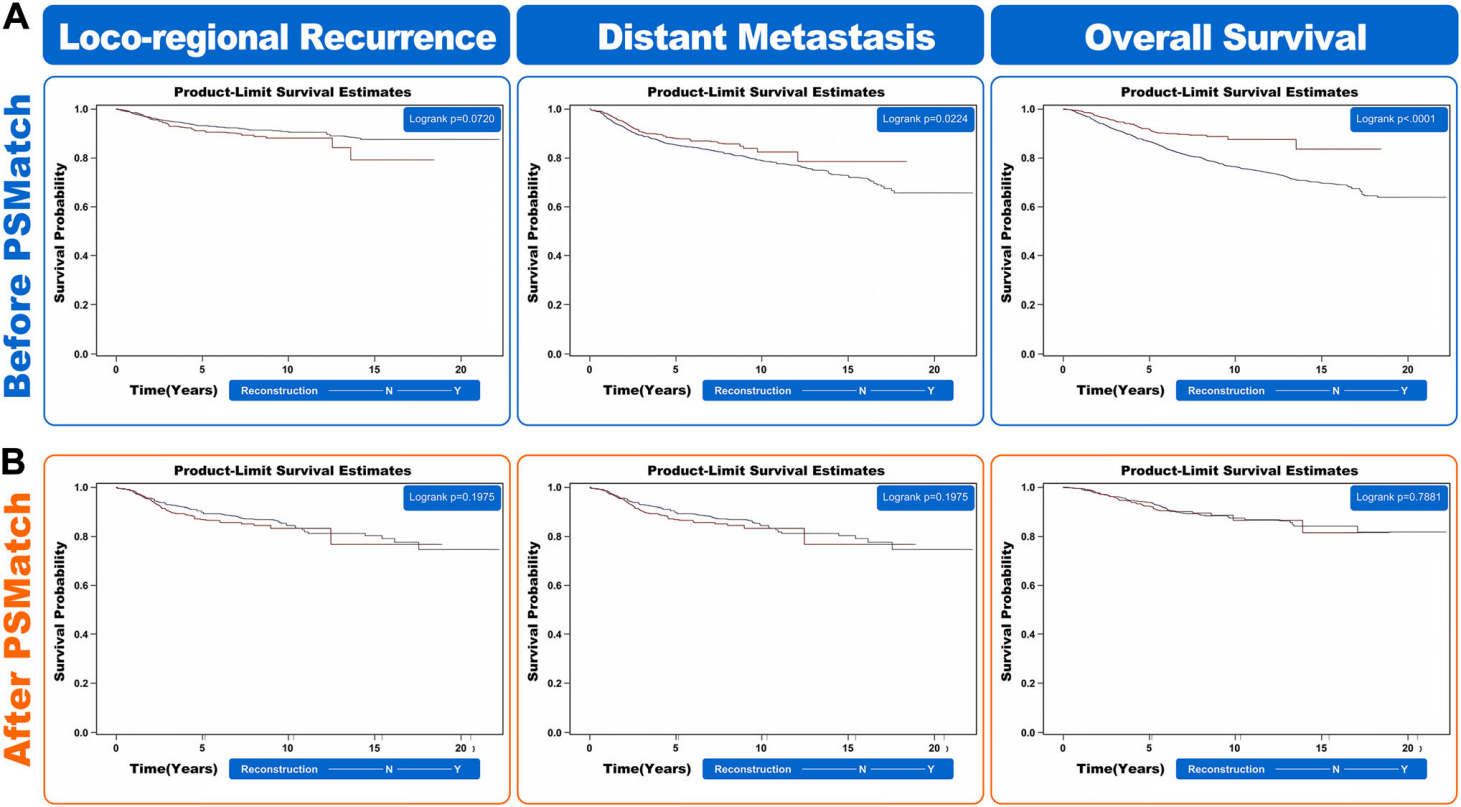
**A** Kaplan-Meier survival curve that evaluates locoregional recurrence, distant metastases, and overall survival in patients with mastectomy alone or mastectomy with immediate breast reconstruction (IBR) before propensity score matching (PSM). **B** Kaplan-Meier survival curve that evaluates locoregional recurrence, distant metastases, and overall survival in patients with mastectomy alone or mastectomy with IBR after PSM

According to the Kaplan–Meier survival curve analysis, there was no difference in local recurrence (*P* = 0.072), but there was less distant metastasis (*P* = 0.022) and better overall survival (*P* < 0.01) in patients who underwent mastectomy and IBR compared with mastectomy alone before PSM (Fig. 3). However, after PSM and with a median follow-up of 106.1 months, there were no differences in locoregional recurrence (*P* = 0.055), distant metastasis (*P* = 0.198) or overall survival (*P* = 0.877) between mastectomy patients with or without breast reconstruction.

## Discussion

Mastectomy usually leaves a stigma of trauma, a reminder of the shadow of breast cancer. IBR has become a popular surgical option for women with breast cancer who are candidates for mastectomy. The procedure offers several advantages over delayed reconstruction, including improved psychological well-being, greater patient satisfaction, and better aesthetic outcomes [5–8]. In the current study, we evaluated the effectiveness and safety of IBR in postmastectomy breast cancer patients.

Our results support the benefits of IBR as we found that patients who underwent this procedure had higher satisfaction rates and improved psychological well-being compared to those who did not undergo breast reconstruction (Table 4). However, there are also potential risks associated with IBR, such as infection and implant loss. It is important for clinicians to weigh the benefits and risks of these procedures on a case-by-case basis and provide appropriate pre-operative counseling to patients.

Implant-based breast reconstruction has gradually replaced autologous breast reconstruction as SSM and NSM have been widely used for mastectomy (Fig. 2). With the advancement of the breast screening program, neoadjuvant chemotherapy, and improvement of surgical techniques, more patients could receive SSM or NSM. In the current study, around 29.4% (1014/3759) of mastectomies were performed with NSM. Recently, the use of endoscopic or robotic-assisted mastectomies has also increased the use of implant-based breast reconstructions [21–23].

Regarding factors promoting breast reconstructions, in the multivariate analysis of mastectomies combined with IBR, age less than 45 years, breast MRI before surgery (OR = 3), luminal A intrinsic subtype, NSM (OR = 8), ORBS (OR = 1.5) and high-volume surgeon (OR = 2.5) were important independent factors contributing to breast reconstruction (Table 3). One of the most important factors related to breast reconstruction is the use of preoperative breast MRI. Preoperative breast MRI was speculated to be associated with increased mastectomy [39]. However, some studies did not support MRI would increase the rate of mastectomy [40, 41]. Studies have shown that preoperative breast MRI in patients who underwent planned mastectomy for multi-centric breast cancer would increase the breast reconstruction rate through a shared decision-making process [14, 39]. We found that ORBS and high-volume breast surgeons had higher rates of breast reconstruction (Table 1 and 3), and these phenomena were also supported in other related studies [42, 43].

One of the most frequently debated questions is whether IBR affects oncologic safety in terms of margin involvement rate, locoregional recurrence, distant metastasis, or overall survival. Oncologic safety is of paramount importance. Propensity score matching (PSM) is a statistical method commonly used to reduce bias [24, 44]. After PSM, no significant differences were observed between the mastectomy with IBR group and the mastectomy alone group in terms of margin involvement rates (4.7% vs. 4.2%, *P* = 0.69), local recurrence (11% vs. 8.3%, *P* = 0.06), distant metastasis (12.6% vs. 13.2%, *P *= 0.71), or overall survival (93.4% vs. 91.1%, *P *= 0.08). With a median follow-up of 106.1 months, Kaplan-Meier survival curve analysis showed no significant difference between patients who underwent mastectomy with or without reconstruction in terms of locoregional recurrence (*P* = 0.055), distant metastasis (*P *= 0.118) or overall survival (*P* = 0.877).

Our current study is limited by its retrospective design, long study period, and insufficient sample size. Despite these limitations, we did enroll 3759 mastectomy patients, with 1091 (29%) undergoing breast reconstructions. The outcomes of patients who underwent mastectomy alone or with IBR were compared, and factors contributing to breast reconstruction were identified. The oncological safety analysis after PSM revealed that mastectomy with IBR did not worsen locoregional recurrence, distant metastasis, or overall survival compared to mastectomy alone. The information derived from the current study was valuable.

## Conclusion

In the current study, we showed that mastectomy with IBR had better patients who reported a cosmetic outcome and comparable oncologic safety compared to mastectomy alone. Independent factors promoting IBR were identified and could help increase the breast reconstruction rate. Clinicians could consider the benefits and risks of the procedure and provide appropriate preoperative counseling to patients.

## Data Availability

The data used in the current study could be provided by request to the principal investigator HWL after acceptance of the manuscript.

## Abbreviations

BCS: Breast-conserving surgery
QoL: Quality of life
SSM: Skin-sparing mastectomy
NSM: Nipple-sparing mastectomy
IBR: Immediate breast reconstruction
CCH: Changhua Christian Hospital
PSM: Propensity score matching
ORBS: Oncoplastic reconstructive breast surgeon
IRB: Institutional Review Board
TRAM: Transverse myocutaneous abdominal
LD: Latissimus dorsi
PROM: Patient-reported outcome measures
MABS: Minimal access breast surgery
OR: Odds ratio
